# Effects of a 3-Year Nurse-Based Case Management in Aged Patients with Acute Myocardial Infarction on Rehospitalisation, Mortality, Risk Factors, Physical Functioning and Mental Health. A Secondary Analysis of the Randomized Controlled KORINNA Study

**DOI:** 10.1371/journal.pone.0116693

**Published:** 2015-03-26

**Authors:** Inge Kirchberger, Matthias Hunger, Björn Stollenwerk, Hildegard Seidl, Katrin Burkhardt, Bernhard Kuch, Christa Meisinger, Rolf Holle

**Affiliations:** 1 Institute of Epidemiology II, Helmholtz Zentrum München, German Research Center for Environmental Health, Neuherberg, Germany; 2 KORA Myocardial Infarction Registry, Central Hospital of Augsburg, Augsburg, Germany; 3 Institute of Health Economics and Health Care Management, Helmholtz Zentrum München, German Research Center for Environmental Health, Neuherberg, Germany; 4 Department of Internal Medicine I—Cardiology, Central Hospital of Augsburg, Augsburg, Germany; 5 Department for Internal Medicine/Cardiology, Donau-Ries-Kliniken, Nördlingen, Germany; McGill University, CANADA

## Abstract

**Background:**

Home-based secondary prevention programs led by nurses have been proposed to facilitate patients’ adjustment to acute myocardial infarction (AMI). The objective of this study was to conduct secondary analyses of the three-year follow-up of a nurse-based case management for elderly patients discharged from hospital after an AMI.

**Methods:**

In a single-centre randomized two-armed parallel group trial of hospitalized patients with AMI ≥65 years, patients hospitalized between September 2008 and May 2010 in the Hospital of Augsburg, Germany, were randomly assigned to case management or usual care. The case-management intervention consisted of a nurse-based follow-up for three years including home visits and telephone calls. Study endpoints were time to first unplanned readmission or death, clinical parameters, functional status, depressive symptoms and malnutrition risk. Persons who assessed three-year outcomes and validated readmission data were blinded. The intention-to-treat approach was applied to the statistical analyses which included Cox Proportional Hazards models.

**Results:**

Three hundred forty patients were allocated to receive case-management (n = 168) or usual care (n = 172). During three years, in the intervention group there were 80 first unplanned readmissions and 6 deaths, while the control group had 111first unplanned readmissions and 3 deaths. The intervention did not significantly affect time to first unplanned readmission or death (Hazard Ratio 0.89, 95% confidence interval (CI) 0.67–1.19; p = 0.439), blood pressure, cholesterol level, instrumental activities of daily life (IADL) (only for men), and depressive symptoms. However, patients in the intervention group had a significantly better functional status, as assessed by the HAQ Disability Index, IADL (only for women), and hand grip strength, and better SCREEN-II malnutrition risk scores than patients in the control group.

**Conclusions:**

A nurse-based management among elderly patients with AMI did not significantly affect time to unplanned readmissions or death during a three-year follow-up. However, the results indicate that functional status and malnutrition risk can be improved.

**Trial registration:**

Current Controlled Trials ISRCTN02893746

## Introduction

Treatment and health care of elderly patients with acute myocardial infarction (AMI) is a still insufficiently investigated field of research. This is surprising since AMI is a disease which mainly affects elderly persons and most of the persons who die from ischemic heart disease are ≥ 65 years old [1]. An increase in AMI cases is expected in the next decade because people aged ≥ 65 years will increase from 92 million in 2013 to 147 million by 2050, representing approximately one third of the projected total population of the European Union countries [2]. This trend is associated with a high financial burden on the countries’ health care system. In addition, elderly patients with AMI often have comorbidities which considerably contribute to polypharmacy, impairments in quality of life, disability and high hospital readmission rates [3].

The transition from hospital discharge to post-discharge care is a critical phase specifically for elderly AMI patients. Since the time spent in the acute hospital has considerably decreased in the last years, elderly patients with AMI often face unmet information needs [4]. Secondary prevention, however, suggests lifestyle changes, increase of physical activity, weight management, risk factor control and pharmacological therapy. A prerequisite of successful secondary prevention is an intensively educated and motivated patient [5]. Implementation of secondary prevention measures may be more difficult for elderly patients due to multimorbidity, reduced social support, and functional or cognitive impairments. Cardiac rehabilitation is expected to bridge the gap between acute in-hospital and long-term out-patient care. In Germany, it is mainly offered as 3-week treatment in specialized rehabilitation hospitals [6]. However, only about 50% of the German patients with AMI receive rehabilitation measures and data indicate that patients older than 60 years are less likely to receive rehabilitation compared with younger patients. The association between older age and non-referral to cardiac rehabilitation is also known in other countries [7]. As compared to other countries, in Germany no home-based post-discharge programs are currently available. Although a number of studies have reported positive effects of nurse-based case-management programs on hospital readmission and other outcomes in CHD, only a few studies have included persons with AMI older than 65 years. Moreover, studies which treat and follow patients longer than one year are scarce [8–10].

The present KORINNA trial was initially planned with an intervention lasting one year [11]. The one-year case-management intervention in patients with AMI aged 65 years or older significantly improved blood lipid levels, functional status and malnutrition risk, but failed to show a positive effect on time to first unplanned readmission or death, which was the primary study endpoint [12, 13]. However, Kaplan-Meier curves indicated that the differences between intervention and control group regarding time to first unplanned readmission or death became larger with increasing observation time. Since available studies on effects of case management in patients with CHD are missing conclusions regarding an optimal length of intervention and follow-up period, it was decided to prolong the study and deliver intervention and follow-up for 3 years in all. Thus, the overall objective of this paper is to conduct secondary analyses of trial data with longer follow-up. Specifically, the aims are to analyze the effect of a 3-year case management intervention led by trained nurses on the time to first unplanned readmission or death and on risk factor profiles, physical functioning and mental health in patients with AMI aged 65 years or older and to determine if the results at 1 year were maintained at 3 years.

## Methods

The protocol for this trial and supporting CONSORT checklist are available as supporting information; see S1 CONSORT Checklist and S1 Protocol.

### Trial design

The KORINNA (‘Coronary infarction follow-up in the elderly’) was a single centre randomized two-arm parallel group trial of patients with AMI who were 75 years or older. The allocation rate was 1:1.

The initial study (1-year intervention and follow-up) was approved by the Ethics Committee at the Bavarian Chamber of Physicians (Reference number: 08064; preliminary approval 28.07.2008; final approval 11.11.2008) and was conducted according to German privacy law and in compliance with the Declaration of Helsinki. The patients gave written informed consent before study inclusion. The ethics committee, which approved the 1-year study, did not require informed consent for the extension of the study to the 3-year intervention and follow-up.

The trial was registered in the Current Controlled Trials database (ISRCTN02893746; http://www.controlled-trials.com/ISRCTN02893746/KORINNA). Since the trial was part of a research consortium involving a number of studies and investigators, the project coordination took longer than expected and resulted in a delayed trial registration on 10.02.2009. The authors confirm that all ongoing and related trials for this intervention are registered.

Patients were recruited from 08.09.2008 to 11.05.2010 and 3-year follow-up examinations took place from 27.09.2011 to 19.06.2013. Since an unexpected low number of older eligible patients became apparent in the first year of recruitment, the study protocol was modified in accordance with the study’s Advisory Board. It was decided to decrease the minimum age of participants from 75 to 65 years. The recruitment phase was expanded accordingly.

Eligible participants were patients ≥ 65 years of age who were hospitalized with a first or recurrent AMI from September 2008 to May 2010 in the Hospital of Augsburg. Patients who lived in institutionalized care or planned to move to it were excluded. In addition, persons who planned to move outside the study region were not included, and patients with severe comorbidity associated with a life expectancy less than one year (e.g., terminal cancer), patients who were unable or unwilling to give written informed consent (e.g., patients with dementia), and patients who had insufficient knowledge of the German language were excluded.

### Interventions

The complex intervention applied in this study is characterized by a combination of components from case-management and disease-management. The identification of individual care problems and the coordination of health care measures is an important component of case-management, whereas support of risk factor management, information and individual education are typical elements of disease-management programs. The development of the intervention was based on reported experiences with nurse-based follow-up programs in patients with heart failure [14] and AMI [15]. Stewart and Blue [14] provided a guideline for researchers to facilitate the planning and conduct of nurse-based intervention studies. The present study adapted their proposals for the application in a study with elderly AMI patients instead of patients with heart failure. The selected main intervention components include identification and management of heart failure symptoms (dyspnea, oedema, liquid and body weight control), symptoms of angina, falls, blood pressure, heart rate, blood glucose, medication and medication adherence, depressed mood, and general physical condition.

Using a structured interview guide, information on the intervention areas was collected by the study nurse, the need for an intervention was estimated and the intervention type (e.g., referral to the general practitioner, education on medication use, contacts to cardiac sports group) was determined. For example, if a patient reported that he/she fell down, the nurse may detect trip hazards in the home environment and may advise on its modification, or may refer to the general practitioner in order to identify possible adverse medication effects. The content of the intervention was individualized insofar as the nurse selected the most appropriate intervention for the individual.

Standard operating procedures have been developed by the multidisciplinary study team, defining the standards to conduct home visits and telephone calls including potential interventions for each area. The study nurses had training on assessments and interventions led by the study physician and the principle investigators. In a 4-week pilot phase, the intervention was tested in 11 patients and was slightly revised. Over the entire study course, a member of the study team attended selected intervention sessions in order to check guideline adherence and comparability of the applied interventions among the study nurses. Emerging problems regarding assessment, intervention or standardization were addressed in regular meetings among study nurses and team members.

Home visits and telephone calls had similar structure and content, but differed regarding location and type of contact between patient and study nurse, and the possibility to perform specific measurements e.g. of blood pressure or blood glucose. Patients assigned to the control group received usual care. Besides care provided by cardiologists and general practitioners, patients may have received in-hospital cardiac rehabilitation or may have participated in disease-management programs offered by German health insurance companies.

**Fig 1:**
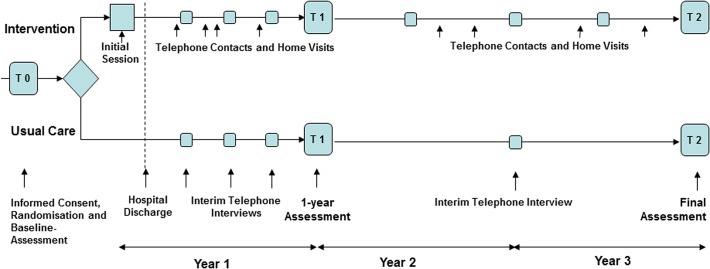
Study design.

Fig. 1 outlines the study design. After giving informed consent, all patients received a baseline assessment in the hospital. Patients randomized into the intervention group received the first intervention (including oral and written information about AMI and comorbidities, medication, recommendations regarding nutrition, smoking, and physical activity) shortly before discharge. A first home visit or telephone call was scheduled 7 to 14 days after discharge. The minimum frequency of interventions delivered by telephone calls was four in the first year (every 3 months), and two in each of the following two years (every 6 months). The decision to reduce the intervention frequency in the second and third year was based on the consideration that the intervention should be affordable to facilitate a future implementation of the case management program in usual health care. For patients who had an unplanned readmission to hospital (study endpoint) the intervention continued until the end of the study or death. Additional interventions delivered by telephone calls or home visits were carried out according to patient need and patient risk level. The risk level was determined according to Russell et al. [16] who suggested a classification based on compliance, social network, and New York Heart (NYHA) classification. For instance, patients with NYHA classification class 1 or 2 who appeared to be compliant and showed good social support at the first home visit, were offered only telephone calls. Patients from the control group were contacted by telephone every 3 months in the first year and once after 2 and 3 years in order to collect data on hospital readmissions and secondary outcomes such as mental and physical health and malnutrition. All patients had an assessment and examination in the hospital after one year and a final assessment after 3 years which was performed at home in most cases.

### Outcomes

The main study outcome was the time between initial hospital discharge and first unplanned readmission to any hospital or death. Only hospital stays with duration of at least 24 hours were considered. All patients were asked for readmissions and acute care visits to physicians, clinics, and ambulatory departments in standardized non-interventional telephone interviews at 3, 6, 9, 12, 24 and 36 months after index hospital discharge. In detail, patients were asked whether they were readmitted to hospital in the past three (or twelve) months, how often they were readmitted, how long they stayed in the hospital, for which reason they were admitted, whether the admission was planned or unplanned, and what was the exact date of admission. Each self-reported readmission was validated by hospital records by a member of the study team who was blinded towards the patients’ group assignment. The person in charge of the hospital validation received a list of the patients’ name and study identification number. For patients who reported a readmission to the Augsburg Hospital, this admission list was screened for the patients’ name and all documented admissions were collected. For patients who reported an admission in any other hospital, the respective hospital was contacted, received the patients’ name and consent and was requested to report all admissions of the study participant during the study course. In order to detect unreported hospital admissions, all hospital admissions at the Augsburg Hospital during the entire study course were screened for all study participants, as well as the admission data of all hospitals in which the study participant was admitted at least once. Information on all-cause mortality was collected based on death certificates obtained from the local health departments.

Additional study endpoints included clinical parameters, functional status, malnutrition risk, cognitive functioning, and depression. Most of them were assessed at baseline, one and three years after discharge (for details see Table 1). Since the 1-year assessment indicated a considerable high respondent burden, we decided to reduce the number of questionnaires for the 3-year examination. We excluded those questionnaires which were specifically exhausting for the patients (e.g. Mini Mental State Exam, Social Support Questionnaire) or measure a health dimension which was already covered by other questionnaires (e.g. Barthel Index which assesses functioning). The Timed up and go test for mobility was not scheduled for the three-year final examination since the 1-year examination showed that most examinations took place at home, where a standardized application of this test is impossible. Baseline blood pressure was obtained from the patients’ medical records. Blood pressure one and three years post discharge was assessed during an examination in the hospital or at home after five minutes of sitting rest. Cholesterol levels were determined from non-fasting venous blood samples collected at follow-up examinations.

**Table 1:** Outcome measures.

Category	Instrument	Months after discharge						
		0	3	6	9	12	24	36
Anamnesis	Baseline questionnaire	●						
Anamnesis	Geriatric Assessment	●						
Quality of life	EQ-5D	●	●	●	●	●	●	●
Quality of life	WHO-5 Well Being Index (WHO-5)	●				●		
Cognitive functions	Mini Mental State Exam (MMSE)	●				●		
Functioning	Barthel Index	●				●		
Functioning	Instrumental Activities of Daily Living Scale (IADL)	●						●
Functioning	Health Assessment Questionnaire (HAQ-DI)	●				●		●
Functioning	Hand grip strength measurement	●				●		●
Mobility	Timed up and go (TUG)	●				●		
Nutrition	Seniors in the Community Risk Evaluation for Eating and Nutrition (SCREEN II, Version II)	●				●	●	●
Social Support	Fragebogen zur sozialen Unterstützung (F-sozU)	●				●		
Depression	Geriatric depression scale (GDS)	●				●		●
Clinical parameters	Blood pressure	●				●		●
	Cholesterol					●		●
Resource use	Questionnaire	●	●	●	●	●	●	●

Functional status was assessed using three different instruments. The Health Assessment Questionnaire Disability Index (HAQ-DI) is a 20-item questionnaire with eight domains assessing functional ability over the past week [17]. Three item response options ranging between 0 (without any difficulty) and 3 (unable to do) are available. The highest item score in each domain determines the domain score and the HAQ-DI total score is the average score of all domains. Further, the Instrumental Activities of Daily Life Scale (IADL) was applied, which measures independent living skills with 8 items [18]. The IADL summary score ranges from 0 (low function, dependent) to 8 (high function, independent) for women, and 0 to 5 for men. Finally, hand grip strength was determined as an indicator of overall muscle strength [19]. It was assessed using a JAMAR hydraulic hand dynamometer (Saehan Corp. Masan, Korea). Three trials with each hand were requested and the maximum value for the right and the left hand, respectively, was used.

Malnutrition risk was assessed using the eight-item version of the SCREEN-II (Seniors in the Community: Risk evaluation for eating and nutrition, Version II) questionnaire [20]. SCREEN-II scores range between 0 (highest risk) to 48 (lowest risk). Depressive symptoms were assessed by the 15-item version of the Geriatric Depression Scale (GDS-15) [21]. Response options are yes/no and the summary score ranges between 0 (no depression) to 15 (high depression).

### Sample size

Sample size estimation was based on the primary study endpoint (hospital readmission or death) one year after discharge. According to a published study with 70-years old patients an event rate of 40% in the control group was assumed [15]. The study was designed to detect an improved rate of 25% in the intervention group (i.e. Δ = 0.15) with a power of 80% and a two-sided type I error level of 5%, this translates to a hazard ratio of 0.56 under the assumption of exponential survival distributions. At least 152 participants per group were needed. A maximum drop-out rate of 10% during the 1-year follow-up period was expected. Thus, it was decided to include a total of 338 participants.

### Randomization

The randomization procedure used randomized blocks within strata for sex, age (< 70 vs. 70–79 vs. 80+ years) and number of comorbidities (diabetes and chronic heart failure) in order to achieve a balanced distribution of these prognostic factors among the treatment groups. In order to ensure the concealment of the allocation, patients were randomized by the biostatistical center of the Helmholtz Zentrum München. Blinding of patients was not possible since home visits were only performed in patients from the intervention group. However, the persons who assessed 1- and 3-year outcomes and the person who validated data on readmissions were not involved in the delivery of interventions and remained blinded towards the patients’ group assignment.

### Statistical methods

Analyses were performed according to the intention-to-treat approach. Differences between the intervention and the control group regarding the time to first unplanned hospital readmission or death were displayed using Kaplan Meier survival curves. Cox proportional hazards regression models were calculated to estimate the intervention effect. Diabetes, heart failure, age and sex were included as independent variables.

To estimate the treatment effect on clinical parameters, functional status, malnutrition risk, cognitive functioning, and depression, we used linear mixed models. Mixed models are a repeated measurement method that can deal with different numbers of observations per subject. They do not exclude persons with missing 12 or 36 month data but use the available information before dropout to estimate means and covariances—thus respecting the intention-to-treat principle [22]. By accounting for within-patient correlations, appropriate adjustments for parameter estimates are made at times when data are incomplete [23]. In contrast to a complete case analysis, mixed models do not require the unrealistic assumption that missingness occurs completely at random (MCAR) in order to provide valid estimates. Instead, only the more plausible “missing at random” assumption is required, where missingness is assumed to be conditionally independent from the unobserved value given the covariates and the observed values before dropout [22, 23].

The intervention effect for each secondary outcome measure was determined as the difference in mean scores between intervention and control group three years after the index hospitalization, as estimated from a linear mixed model with treatment x time interaction. As in clinical trials using stratified randomization procedures, all subsequent analyses must be adjusted by the stratification variables to give valid inference, we included sex, age and number of comorbidities as additional covariates [24].

In general, each mixed model used data from baseline and the 12 and 36 month measurements to estimate the treatment effect at the end of the study, with the following exceptions: As no blood sample was collected at baseline, estimations for lipid parameters use data from 12 and 36 month measurements only. Also, blood values after 36 months are only available for 182 patients. The IADL scale was not administered at 12 months so that estimation uses baseline and 36 months data only. Also, as IADL scores are calculated differently for women and men, we present results stratified by sex [18]. The SCREEN-II questionnaire was also administered in an additional telephone interview at 24 months so that estimation is based on data collected at annual intervals.

## Results

**Fig 2:**
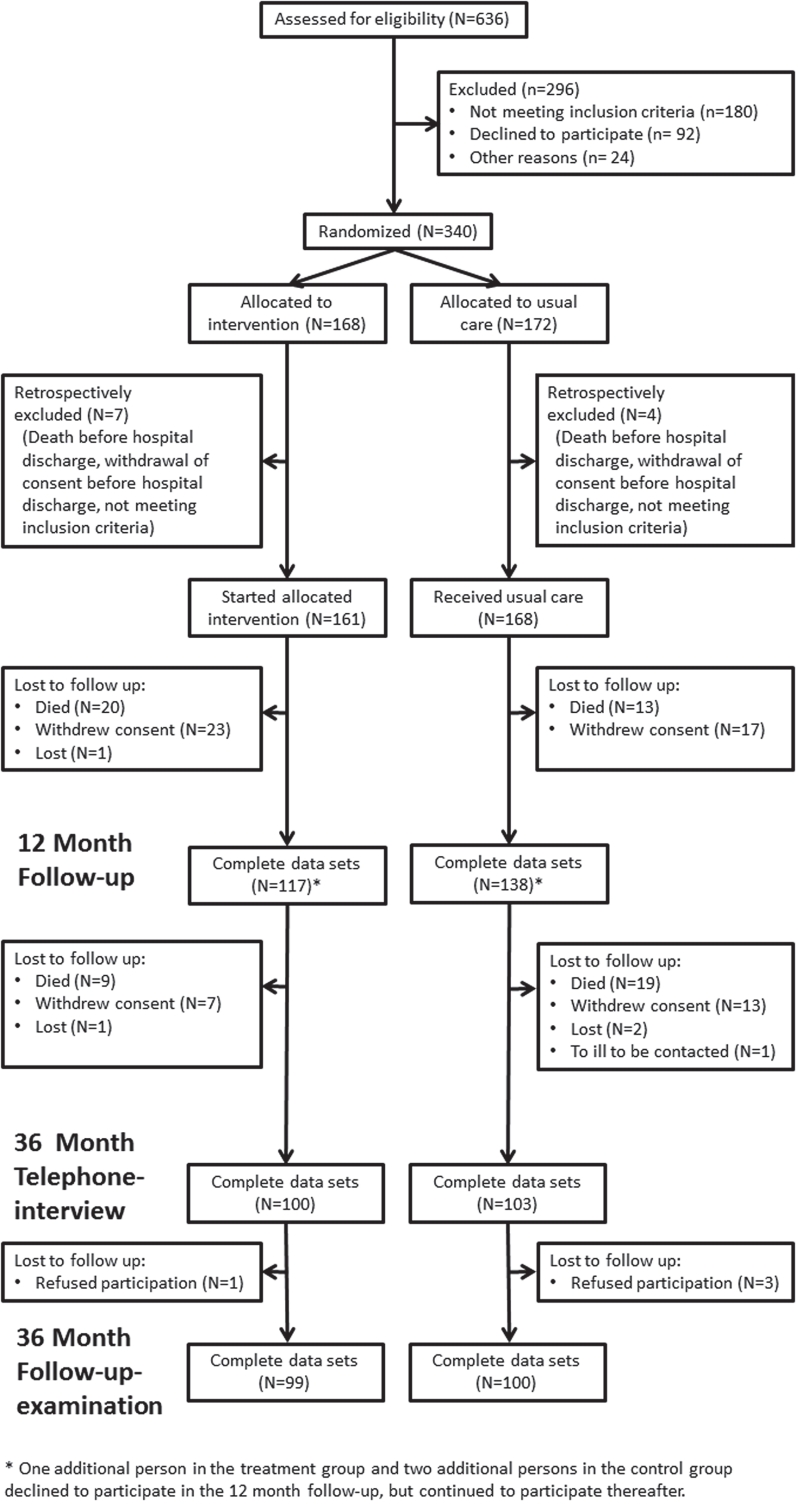
Flow of participants through the KORINNA trial.

From the 636 patients who were initially screened for participation, 161 patients started the allocated intervention and 168 persons received usual care. Fig. 2 details the flow of participants through the study course. Table 2 displays the baseline characteristics of these 329 participants. Intervention and control group were similar with the exception of diabetes which was more common in the control group.

**Table 2:** Patient characteristics at baseline.

Characteristic	Intervention group (n = 161)	Control group (n = 168)
**Sociodemographic characteristics**		
Mean age, years ± SD	75.2 ± 6.0	75.6 ± 6.0
Male sex, n (%)	101 (62.7)	103 (61.3)
Living alone^a^, n (%)	42 (26.8)	44 (26.4)
School education^a^ > 9 years, n (%)	45 (29.6)	32 (19.4)
**Risk factors and comorbidities**		
Diabetes mellitus, n (%)	45 (28.0)	61 (36.3)
Congestive heart failure, n (%)	48 (29.8)	47 (28.0)
Blood pressure, mean ± SD ^a^		
Systolic BP, mmHg	121.6 ± 13.7	124.2 ± 13.5
Diastolic BP, mmHg	71.4 ± 7.8	71.3 ± 8.3
Hyperlipidaemia, n (%)	79 (49.1)	84 (50.0)
BMI (kg/m^2^), mean (SD)*	27.7 ± 4.2	27.3 ± 3.9
Smoking^a^, n (%)		
Current smoker	21 (13.6)	19 (11.5)
Ex-smoker	76 (49.4)	67 (40.4)
Never-smoker	57 (37.0)	80 (48.2)
**AMI characteristics and treatment, n (%)**		
Re-infarction	33 (20.5)	41 (24.4)
ST-segment elevation MI	68 (43.6)	50 (30.3)
Non-ST-segment elevation MI	71 (45.5)	95 (57.6)
Bundle branch block	17 (10.9)	20 (12.1)
Any reperfusion treatment	140 (87.0)	141 (83.9)
Coronary artery bypass grafting	26 (16.2)	24 (14.3)
PCI without stenting	4 (2.5)	9 (5.4)
PCI with stenting	112 (69.6)	113 (67.3)
Any in-hospital complication ^a^ ^,^ ^b^	31 (19.5)	26 (15.5)
Cardiac rehabilitation in the first 3 months^a^	68 (54.4)	84 (52.8)
**Physical/mental health,** mean ± SD^a^		
HAQ-DI Score	0.8 ± 0.8	0.8 ± 0.8
IADL-Score	5.6 ± 1.6	5.6 ± 1.5
MMST	26.7 ± 4.1	26.4 ± 3.8
GDS	3.2 ± 3.1	3.2 ± 2.6
Social support	3.9 ± 0.6	3.9 ± 0.6
WHO-5-Well-Being Index	13.6 ± 6.8	13.2± 6.9

^a^ n (%) refer to non-missing observations

^b^ Includes pulmonary oedema, re-infarction, bradycardia, ventricular tachycardia and cardiogenic shock

*BMI: Body Mass Index

From the 161patients in the intervention group, 132 (82%) had a first home visit. The number of home visits in the first, second and third year of follow-up were 186, 4 and 1, respectively. Home visits had a mean duration of 117 minutes including a mean traveling time of about 35 minutes. Moreover, patients from the intervention group had 489 telephone calls in the first year, 213 in the second, and 204 in the third year of follow-up. A telephone interview lasted 19 minutes in average. During the entire follow-up period patients had in average 1.2 home visits and 5.6 telephone sessions.

During follow-up, 200 participants (86 in the intervention group, 114 in the control group) had an event (i.e. first unplanned readmission or death). In the intervention group 80 of the events were first unplanned readmissions and 6 were deaths, whereas in the control group 111 of the events were first unplanned readmissions and 3 were deaths. In addition, 23 patients in the intervention group and 29 patients in the control group of those with unplanned readmissions died within the follow-up period.

Overall, during the entire follow-up period, 422 unplanned admissions and 226 planned admissions to hospitals were registered. Among these, there were 160 unplanned and 118 planned hospital stays in the intervention group, and 262 unplanned and 108 planned hospital stays in the control group. 44 patients in the control group and 55 patients in the intervention group had no hospital stay during the three-year follow-up.

**Fig 3:**
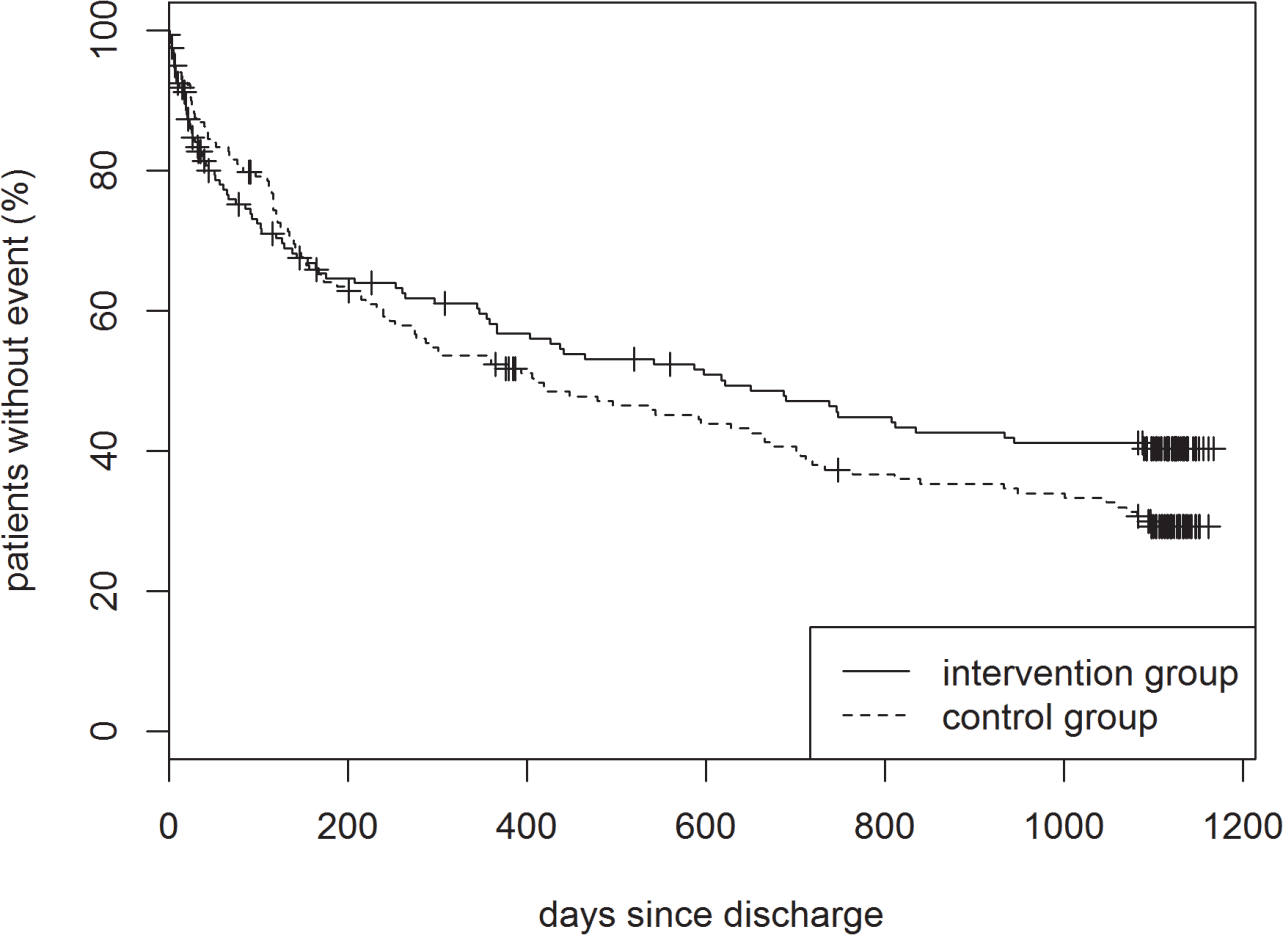
Kaplan Meier curve until first unplanned readmission to hospital or death (total sample). LogRank test: p = 0.1424.

The Kaplan-Meier curves for the combined end point of unplanned readmissions and death are displayed in Fig. 3. Cox regression analysis revealed no significant effect (HR 0.89, 95% CI 0.67–1.19; p = 0.439) (see Table 3). As can be seen from the crossing Kaplan-Meier curves, the proportional hazards assumption may be violated which is confirmed by finding a significant treatment*time interaction term. However, we decided against choosing a different test because this would not be a confirmatory analysis in the strict sense.

**Table 3:** Results of the Cox regression model for the primary outcome unplanned hospital readmission or death.

Parameter	Estimate	Standard error	Hazard ratio	95% confidence interval	P value
Intervention group	-0.11	0.14	0.89	0.67–1.19	0.439
Diabetes	0.39	0.15	1.47	1.09–1.99	0.012
Heart failure	0.25	0.16	1.28	0.94–1.75	0.128
Age	0.06	0.01	1.06	1.04–1.09	<.001
Male sex	-0.01	0.16	0.99	0.73–1.35	0.976

The mean scores of the outcome measures addressing clinical parameters, functional status, malnutrition risk, cognitive functioning, and depression in the two treatment arms at three year and the respective adjusted mean differences are displayed in Table 4. No significant differences between intervention and control group were found for any of the clinical parameters and for the IADL scores among men. However, there were significant effects of the intervention on functional status. Patients in the intervention group had significantly more favorable HAQ-DI scores (-0.24, 95% CI -0.41 to-0.07; p = 0.007) and better hand grip strength (2.15, 95% CI 0.17 to 4.14; p = 0.033) than persons in the control group. In addition, among women, patients in the intervention group had better IADL scores (0.85, 95% CI 0.24 to 1.47; p = 0.007) compared with persons in the control group. Analyses also indicated a beneficial intervention effect on malnutrition risk as assessed by SCREEN-II (2.69, 95% CI 0.94 to 4.44; p = 0.003.)

**Table 4:** Mean values for clinical parameters, physical functioning and mental health scores at three years.

Outcome	Intervention Group		Control Group		Difference adjusted by stratification variables (Mixed Model)	
	N	Mean (SD)	N	Mean (SD)	Mean (95% CI)	p-value
**Clinical parameters**						
Systolic blood pressure, mmHg^a^	99	136.7 (20.9)	98	140.9 (20.4)	-1.11 (-6.59 to 4.37)	0.691
Diastolic blood pressure, mmHg^a^	99	75.4 (11.9)	98	76.7 (10.9)	-1.50 (-4.78 to 1.78)	0.369
LDL cholesterol, mg/dl^a^ ^,^ ^b^	92	94.0 (29.0)	90	101.7 (35.1)	-8.52 (-17.2 to 0.12)	0.053
Total cholesterol/HDL cholesterol, mg/dl^a^ ^,^ ^b^	92	3.76 (1.19)	90	3.72 (1.12)	-0.07 (-0.34 to 0.28)	0.846
**Physical functioning/mental health**						
HAQ-DI Score^a^	99	0.57 (0.68)	100	0.85 (0.86)	-0.24 (-0.41 to-0.07)	0.007
Hand grip strength, kg	99	30.6 (10.3)	96	26.7 (11.1)	2.15 (0.17 to 4.14)	0.033
IADL Men	71	4.69 (0.80)	61	4.48 (1.12)	0.18 (-0.12 to 0.49)	0.231
Women	28	7.46 (1.04)	39	6.36 (2.22)	0.85 (0.24 to 1.47)	0.007
SCREEN-II	100	39.4 (5.9)	103	36.7 (7.3)	2.69 (0.94 to 4.44)	0.003
GDS^a^	99	2.61 (3.07)	99	3.37 (3.22)	-0.44 (-1.08 to 0.20)	0.176

SD, standard deviation

CI, confidence interval

LDL, Low-density lipoprotein

HDL, High-density lipoprotein

HAQ-DI, Health Assessment Questionnaire Disability Index

IADL, Instrumental Activities of Daily Living Scale

GDS, Geriatric Depression Scale

SCREEN-II, *Seniors in the Community*: *Risk evaluation for eating and nutrition*, *Version II*

^a^Smaller values are better

^b^Data available for 182 patients

## Discussion

The present study failed to find a significant beneficial effect of a 3-year nurse-based case management intervention on the time to first unplanned readmission or death within three years after discharge in elderly patients with AMI as compared with usual care. However, a significant beneficial intervention effect could be shown for functional status as assessed by HAQ-DI, IADL (only for women), and hand grip strength, and for malnutrition risk (SCREEN-II).

Compared with the results of the 1-year follow-up, results in terms of unplanned readmissions/death, hand grip strength and malnutrition were maintained after three years [12,13]. Differences between intervention and control group regarding HAQ-DI and IADL (for women) increased and reached statistical significance after three years. In contrast, the finding that the patients in the intervention group had significantly better low density lipoprotein cholesterol levels at the 1-year follow-up could not be confirmed after three years.

Overall, it is difficult to compare the results of the present study with prior studies. Studies are lacking comparability regarding study design, nature and duration of intervention, duration of follow-up, study endpoints and country of origin [8, 9, 25]. To our knowledge, the present study is the first which included exclusively patients with AMI ≥ 65 years and investigated the effects of a nurse-based intervention with a 3-year duration and follow-up. Other studies on patients post-AMI have also included younger patients, had a shorter intervention and follow-up period and did not provide both home visits and telephone interventions [26, 27].

In terms of the intervention effect on time to first readmission or death, the results of the present study are contrary to a number of studies which reported a benefit of nurse-led secondary prevention programs in persons with CHD or acute coronary syndrome on survival or hospital readmissions [8, 25, 28]. On the other hand, a review including 12 randomized trials performed in patients with established CHD which investigated the influence of case management on the process of care and mortality showed that case management significantly reduced admissions to hospital but failed to show a survival benefit [9]. Most of these studies, however, have included patients < 65 years, and some have even excluded patients > 80 years [28]. Only a few studies were restricted to patients >65 years [29, 30]. For instance, Naylor et al. [29] examined whether a comprehensive discharge planning and home follow-up intervention in elderly hospitalized persons with different health conditions has an effect on outcomes. It could be demonstrated that such an intervention can reduce readmissions and lengthen the time between discharge and readmission. Strandberg et al. [30] conducted a study with patients with cardiovascular disease (40% AMI) ≥ 75 years with an intervention based on consultations by geriatricians and found no significant differences regarding mortality between intervention and control group after 3.4 years of follow-up.

The KORINNA study revealed significant beneficial intervention effects on functional status and risk of malnutrition. The positive effect on the HAQ-DI can be regarded as a clinically important effect since the minimally important difference ranges between 0.10 and 0.22 [17]. The difference between intervention and control group with respect to hand grip strength was significant, however, with 2.5 kg it was smaller than the minimally important difference, estimated to be around 5.0 kg [31]. Overall, the beneficial intervention effect on functional status is in accordance with a number of previous publications which, however, often had a shorter follow-up period, used different outcome measures and/or have included younger patients [10, 26, 29, 32–34]. For elderly patients, Hanssen [26,27] found differences in the physical summary component scale from the Short Form-36 Health Survey in favor of the intervention group after 18-month only in patients older than 70 years, not in younger individuals. In contrast, the study of Naylor et al. [10] exclusively included patients aged 65 years or older, but found no significant improvements in physical health in the intervention group after one year.

Furthermore, the results of the KORINNA study suggest that the intervention has significantly reduced the risk of malnutrition. There exist only a few studies which have assessed nutrition, one of these also showed a significant beneficial effect of the corresponding telehealth intervention [35–37]. However, assessment of nutritional risk in these studies was based on intake of unhealthy food, whereas the SCREEN-II has a focus on risk factor questions, for example about appetite or swallowing difficulties [20].

In terms of LDL cholesterol level, we found a significant difference between intervention and control group after one year [13], but the effect attenuated and was no longer significant after 3 years. This trend is consistent with Redfern et al. [34] who reported a significant beneficial intervention effect after one year, but no significant differences between intervention and control group after 4 years.

The KORINNA study included older patients with AMI who were so far rarely included in randomized controlled trial. Its results indicated that it seems possible to improve functional status and to reduce malnutrition risk in elderly AMI patients by a nurse-based case management intervention. However, during the 3-year follow-up period these improvements were not converted to readmission and survival benefits. This finding is consistent with Strandberg et al. [30] who found significant risk factor reduction but no survival benefits in their 3.4-year follow-up intervention study on patients with cardiovascular disease aged ≥ 75 years. In the KORINNA study, Kaplan-Meier curves for the combined end point of unplanned readmissions and death showed a delayed positive response to the intervention: differences between the intervention and control group became apparent 6 months after discharge and constantly increased up to 3 years. It remains unclear whether a longer follow-up period would have yielded a significant difference between the groups. Perhaps intervention effects have a longer delay in elderly people. In addition, it seems possible that a high number of comorbidities and functional limitations, which are more common in the elderly, hinder the intervention to be as effective as in younger persons. Our finding that the intervention applied in the KORINNA study was beneficial in terms of readmissions and survival among those patients, who had minor functional impairments, would support this hypotheses.

The intervention provided within the KORINNA trial was designed to be individualized regarding content, location and frequency. This approach differs from standard intervention schedules which have been frequently applied in previous trials [8]. A few studies already indicated that individualized interventions may be superior to standard interventions [38, 39]. However, it is not clear whether this also applies to persons in the older age group. In addition, it remains unclear whether the timing of the interventions in the KORINNA study was optimal to maximize their effectiveness. Since a high number of events occurred in the first two months, it could have been useful to have more frequent interventions in this period. Lastly, there is still a lack of knowledge on the most effective types of interventions [25, 40, 41], specifically for elderly patients. Thus, further research on the effectiveness of different interventions schedules is warranted [8, 9, 42].

Finally, it is possible that usual care in the KORINNA trial was already close to the optimum. Treatment and health care provided to the control group probably had a considerable positive effect on health outcomes and therefore the additional benefit of the nurse-based case management may be very small.

The strengths of the KORINNA study refer to the inclusion of AMI patients aged ≥ 65 years and the long-term intervention and follow-up period. The generalizability of the results is therefore limited to elderly patients with AMI. The single center study design is a further possible limitation. Moreover, a number of eligible patients declined to participate prior to randomization or withdrew consent after randomization. Any systematic differences between the excluded patients and the participants would also reduce the external validity of the results. We cannot completely exclude that patients did not report a readmission to a hospital which was not screened for all admissions during the study course.

A nurse-based case-management program could improve post-AMI care in countries such as Germany, where medium- and long-term effects of cardiac rehabilitation, which is offered mainly as an in-patient 3-week treatment, need improvement [6, 43]. In addition, those patients who are commonly not receiving cardiac rehabilitation, e.g. elderly patients or patients with bad health status, may benefit from such a program. A prerequisite for a successful implementation of such a program is its cost-effectiveness. Further research is needed in order to identify most effective intervention types and schedules for elderly patients with AMI. Future trials on the same topic might consider the following points in order to improve study: First, it would be meaningful to apply the intervention only to individuals for whom a specific need was identified. Second, in order to improve the effectiveness of the intervention, the intervention team should consist of a multidisciplinary team including e.g. pharmacists and gerontologists who have a specific knowledge in the pharmacological treatment of the population of elderly, mostly multimorbid patients.

## Supporting Information

S1 Protocol Study protocol. (PDF)

S1 CONSORT Checklist CONSORT 2010 checklist of information to include when reporting a randomized trial. (DOC)
