# Supplementary material for: Effects of a 3-Year Nurse-Based Case Management in Aged Patients with Acute Myocardial Infarction on Rehospitalisation, Mortality, Risk Factors, Physical Functioning and Mental Health. A Secondary Analysis of the Randomized Controlled KORINNA Study
Source: PLoS One. 2015 Mar 26;10(3):e0116693. doi: 10.1371/journal.pone.0116693 (PMC4374800; doi:10.1371/journal.pone.0116693)
Supplement: S1 Protocol — (PDF) [file pone.0116693.s001.pdf]

A Case Management Intervention for Aged Patients With Myocardial Infarction

Koronarinfarkt-Nachbehandlung im Alter (KORINNA)

Study Protocol

Version 1.0

Date: June 04, 2009

1. STUDY SYNOPSIS 4

2. AIM OF THE TRIAL 6

2.1 MEDICAL PROBLEM 6

2.2 EVIDENCE 7

2.3 THE NEED FOR A TRIAL 7

2.4 STUDY RATIONALE 8

3. STUDY DESIGN 8

3.1 STUDY OBJECTIVES 9
3.1.1 Primary objective 9
3.1.2 Secondary objectives 9

3.2 INTERVENTION AND CONTROL GROUP 9
3.2.1 Discharge 9
3.2.2 Telephone contacts 10
3.2.3 Home visits 10
3.2.4 Training and supervision of study nurses 11
3.2.5 Cooperation with GPs 11
3.2.6 Treatment in control group 11

3.3 PATIENT SELECTION 12
3.3.1 Inclusion criteria 12
3.3.2 Exclusion criteria 12

3.4 OUTCOME MEASURES 12
3.4.1 Primary endpoint 12
3.4.2 Secondary endpoints 12

3.5 METHODS AGAINST BIAS 13
3.5.1 Randomization 13
3.5.2 Blinding 13

3.6 PROPOSED SAMPLE SIZE / POWER CALCULATIONS 13
3.6.1 Primary statistical hypothesis 13
3.6.2 Relevant difference and error probabilities 13
3.6.3 Rate of loss to follow up 14
3.6.4 Required sample size 14

3.7 FEASIBILITY OF RECRUITMENT 14
3.7.1 Previous experiences 14
3.7.2 Achievability of recruitment rate 14

4. STATISTICAL ANALYSES 15

4.1 ANALYSIS OF PRIMARY ENDPOINT 15

4.2 INTERIM ANALYSIS 15

4.3 ANALYSIS OF SECONDARY ENDPOINTS 15

4.4 MISSING DATA/ANALYSIS SETS 15

4.5 ECONOMIC ANALYSIS 16

4.6 ANALYSIS PLAN 17

5. ETHICAL CONSIDERATIONS 17

5.1 RANDOMIZATION 17
5.2 INFORMED CONSENT 17
5.3 DATA CONFIDENTIALITY 17
5.4 ETHICAL APPROVAL 18
5.5 PROTOCOL AMENDMENTS 18

6. TRIAL MANAGEMENT 18

6.1 STUDY PROCEDURES 18
6.1.1 Recruitment 18
6.1.2 Randomization 18
6.1.3 Baseline Assessment 19
6.1.4 Interim Assessment 20
6.1.5 Final assessment 20

6.2 QUALITY ASSURANCE/MONITORING 21

6.3 DATA MANAGEMENT 21
6.3.1 Data management in Augsburg 22
6.3.1.1 Organisation-DB 22
6.3.1.2 ACCESS data base 22
6.3.1.3 CATI-data base 22
6.3.1.4 KORA Myocardial Registry data base 22
6.3.2 Data transfer 22
6.3.3 Data security 23
6.3.4 Archiving 23

6.5 TRIAL TIMELINE FLOW 23

6.6 TRIAL TERMINATION 23

7. RESPONSIBILITIES AND TRIAL SUPPORT 23

7.1 TRIAL-SUPPORTING FACILITIES 23
7.2 STEERING COMMITTEE 24
7.3 DATA SAFETY AND MONITORING BOARD 24
7.4 DISSEMINATION AND PUBLICATION OF RESULTS 24

8. REFERENCES 25

1. STUDY SYNOPSIS

**PRINCIPAL INVES-TIGATORS**	**Christa Meisinger, MD, MPH**<br>Augsburg Hospital, MONICA/KORA Myocardial Infarction Registry<br>Stenglinstr. 2, 86156 Augsburg, Germany<br>Tel: ++49/821/400-4373<br>Fax: ++49/821/400-2838<br>christa.meisinger@helmholtz-muenchen.de<br>**Bernhard Kuch, MD**<br>Augsburg Hospital, Department of Internal Medicine I – Cardiology<br>Stenglinstr. 2, 86156 Augsburg, Germany<br>Tel: ++49/821/400-2956<br>Fax: ++49/821/400-3739<br>kuchb@aol.com<br>**Rolf Holle, Prof. Dr.**<br>Helmholtz Zentrum München –<br>German Research Center for Environmental Health<br>Ingolstädter Landstr. 1, 85758 Neuherberg<br>Tel: ++49/89/3187-4192<br>Fax: ++49/89/3187-3375<br>holle@helmholtz-muenchen.de
**TITLE OF STUDY**	**A case management intervention for aged patients with myocardial infarction**
**CONDITION/TOPIC**	Myocardial infarction
**OBJECTIVE(S)**	1. To assess whether a case management intervention by trained nurses can reduce readmission in aged patients with myocardial infarction<br>2. To estimate the cost-utility of this case management intervention
**INTERVENTION (S)**	Experimental intervention: Case management by trained nurse on the basis of telephone contacts and home visits<br>Control intervention: Usual care<br>Duration of intervention per patient: One year
**KEY INCLUSION AND EXCLUSION CRITERIA**	Key inclusion criteria: Patients with acute myocardial infarction, aged 70 years and older, living at home<br>Key exclusion criteria: insufficient command of German language, patients already receiving regular ambulatory home care
**OUTCOME(S)**	Primary efficacy endpoint: Unplanned rehospitalisation (or out of hospital death)<br>Primary economic endpoint: Incremental costs per quality adjusted life year (QALY) gained<br>Key secondary endpoints: Functional status, quality of life, costs, adherence to medication
**STUDY TYPE**	Randomized, observer blind parallel group study
**STATISTICAL ANALYSIS**	Efficacy: time-to-first unplanned readmission (or death) analysed by survival analysis methods (log rank test and/or Cox model)<br>Description of the primary analysis and population: Time to combined endpoint (rehospitalisation or death) will be compared between treatment groups in the intention-to-treat population<br>Economic analysis: estimation of incremental cost-utility ratio with confidence interval based on bootstrap estimation<br>Safety: all occurrences of readmission or death will be monitored regularly<br>Secondary endpoints: analysis by linear and generalized linear models

**SAMPLE SIZE**	To be assessed for eligibility n = 400<br>To be allocated to trial: n = 338<br>To be analysed: n = 302
**TRIAL DURATION**	First patient in to last patient out: 27 months<br>Duration of the entire trial: 36 months (including data cleaning and analysis)
**SUMMARY**	Background: Patients with coronary heart disease (CHD), in particular aged patients, have a high prevalence of co-morbidity associated with poor quality of life, physical disability, high health care costs, multiple medications, and increased risk for adverse outcomes.<br>Objective: In a randomized clinical trial in aged patients (70+ years) with myocardial infarction (MI) it will be evaluated whether a case management intervention by trained nurses will reduce readmission or death, and improve health-related outcomes like functional status, quality of life, and others. To evaluate cost-utility, the additional costs per quality-adjusted life year (QALY) gained will be calculated.<br>Design: Patients will be randomized to either case management intervention or usual care. After discharge from the index hospitalization, standardized telephone interviews as well as home visits will take place as part of the intervention. Twelve months after the index hospitalization patients in both groups will be re-assessed by the study physician. The assessment will be observer-blind and blood samples will be collected.<br>Analysis: Time to first readmission (or out-of-hospital death) will be analyzed by Cox model. The economic analysis will be based on the incremental cost-utility ratio and its confidence interval estimated by bootstrap methods.<br>Consequences: The findings should be used to guide clinicians, administrators and policy makers in the provision of high-quality care to older patients with CHD and comorbidities.
**PARTICIPATING CENTERS**	This is a monocenter study with Klinikum Augsburg as the only recruiting study site. During follow-up, participation of the treating GPs in the region of Augsburg is sought for validation of patient information about events.

2. AIM OF THE TRIAL

2.1 MEDICAL PROBLEM

The aging of the population and the increasing prevalence of chronic diseases imply great challenges to the German health system. Coronary heart disease (CHD) is the leading cause of mortality and morbidity in the industrialized world. The treatment of acute myocardial infarction (MI) has improved dramatically over the last 10 to 20 years and nowadays, aged patients with an acute MI are receiving treatment that had been limited to mainly younger patients about a decade ago [1]. Subsequently, the number of survivors with MI will increase in the next decades.

Patients with CHD, in particular aged patients, have a high prevalence of co-morbidity associated with poor quality of life, physical disability, high health care costs, multiple medications, and increased risk for adverse outcomes. Common co-morbidities among patients with CHD are diabetes, chronic heart failure (CHF), chronic obstructive pulmonary disease (COPD), and depression [1-3]. CHD is the most common reported cause of chronic heart failure in all age-groups [4]. A number of studies also found that more aged than young patients develop CHF following an acute MI: 47% of those aged greater than 75 years versus 23% aged < 75 years [5]. In addition, diabetes is not only a co-morbidity of CHD, but is also an independent risk factor for the development of CHF particularly in the aged [6]. CHF is the most common cause of hospital admission in the aged [7], and more aged than younger patients with CHF are discharged to long-term care and this number is increasing [8]. Thus, as a rule, aged patients do not present with an isolated medical problem, but have multiple co-morbid diseases [9]. Consequently, these patients receive a number of medications [10].

Due to changes in the German health care system, in particular the establishment of the diagnosis related groups (DRG) system, older patients with complex health care needs are nowadays discharged earlier from hospital than a few years ago. For many of these patients an optimal care at home is not guaranteed, so that subsequent readmissions or final admission at a nursing-home result. Medication compliance and lack of medication-related knowledge are serious problems in aged patients, and in higher age-groups there is also often a lack of social support or knowledge to seek medical support promptly, when specific symptoms appear [11]. Thus, optimizing care for this population is a high priority.

Many hospital admissions among aged patients with CHD can be attributed to behavioural and social factors rather than to deteriorating cardiac function or an intercurrent cardiac event [12]. Thus, aged multimorbid patients at risk of poor outcomes might benefit from intensive home follow-up. A nurse-led home-based intervention program including patient education and counselling might improve medication compliance, medication-related knowledge and thus processes of care, coronary risk factor profiles, functional status, and quality of life. As a consequence, this might reduce mortality. In addition, such a program is expected to reduce health care resource use in patients with CHD and consequently total health care expenditures.

A number of prior intervention trials investigated whether a nurse-based case management may influence patient readmission and other outcomes. These studies mostly included patients with CHD younger than 70 years [13] or included persons hospitalized with one of several common medical and surgical reasons [14, 15]. So far, no intervention trial focused on a specific case management programme for the group of aged patients (70+ years) after MI. Case management focuses on delivering personalized services to patients to improve their care. Case management after hospital discharge could play a central role for the prevention of re-infarction or readmission for other reasons among aged patients with an acute MI.

The aim of the planned intervention trial is to examine the effectiveness of a nurse-based case management in patients aged 70 years and older discharged after treatment of an

acute MI in hospital. We hypothesize that such an intervention may influence patient readmission and other outcomes like quality of life and long-term mortality, and thus induce higher cost-effectiveness.

2.2 EVIDENCE

Several randomized trials on case management programmes in secondary prevention of CHD have been published [13, 16], but only very few of the studies were performed in patient groups with a mean age above 70 years. In a meta-analysis investigating the effectiveness of secondary prevention programs with and without exercise components 63 randomized trials were included [13]. Almost all of the included studies were conducted in patients with CHD younger than 70 years. Although this meta-analysis showed that secondary prevention programmes positively affect process of care, survival, and functional status or quality of life for patients with CHD independent of the applied program, these findings can not be generalized to higher age-groups.

In another review McAlister et al. examined whether case management programmes for patients with established CHD improve process of care and reduce mortality [16]. Altogether 12 randomised trials also including mainly patients younger than 70 years could be identified by the investigators. It could be shown that comprehensive case management programmes have a positive effect on processes of care in patients with CHD: there was a significant reduction in admissions to hospital and an improvement in quality of life. However, these randomized clinical trials failed to document any survival benefit or reduction in recurrent myocardial infarction.

Furthermore, only three of these trials described the costs of the intervention. Two reported that their intervention was cost saving but none performed formal cost-effectiveness analyses. The study by Naylor et al [17] examined a nurse-centered discharge planning and home follow-up intervention in patients 65+ (mean age 75 years) with a broad variation of diagnoses and interventions (CHD, CHF >50%). Time to first readmission was increased and there were substantial cost savings (both $p < 0.001$).

2.3 THE NEED FOR A TRIAL

The fourth report on the situation of the aged generation presented by the German Federal Ministry of Family Affairs, Senior Citizens, Women and Youth in 2002 points at specific needs for intensified research activities. One of the major points is the lack of methodologically sound intervention studies in the higher age group [18]. Many drug-related randomized clinical trials exclude high age patients because of methodological reasons [19].

The results of similar intervention trials show that nurse-based case management may influence patient readmission and other outcomes. However, there is no specific programme for the group of aged patients after MI which is tailored to their needs. Since acute intervention has changed for these patients over the last decade, new strategies for secondary prevention in this group have to be established in order to maintain the initially successful cardiac intervention.

There are relatively few non-drug intervention studies in secondary prevention taking place in the ambulatory sector in Germany. Health care research at the intersection of inpatient and outpatient care faces many difficulties and often lacks adequate sponsoring. The planned trial will provide a good opportunity to generate evidence based data on new models for case management and patient support in Germany. The economic component of the study is important in view of scarce resources and possible cost savings as shown by similar studies in other countries.

2.4 STUDY RATIONALE

Patients 70 years and older will be included in this study because prior studies have found, that 55% of aged patients with CHD have two or more secondary diagnoses [20, 21] causing the prescription of complex medication regimes. Common comorbidities in this age group are in particular diabetes and CHF [22]. Diabetes is present in 20 to 35 % [23] and CHF in up to 60% of aged patients with CHD [12, 23]. These conditions do not only coexist, but they can also precipitate frequent admission to hospital in this age. Furthermore, we chose this age group because it can be hypothesized that usual care is suboptimal in aged multimorbid persons. Thus, these patients would extraordinarily profit from the planned intervention.

We chose the primary composite endpoint of unplanned readmission and out-of-hospital mortality. Prior randomized clinical trials in patients with CHF, which chose this endpoint, could show that a home follow-up intervention by a study-nurse reduced unplanned readmissions [24]. This endpoint is of importance from the patient's perspective and from the economical perspective. Further relevant secondary outcomes are those measures, which will be supervised regularly in the intervention group. Among these are the compliance with medication, adjustments of blood glucose values and hemoglobin-A_{1c} in patients with type 2 diabetes as well as the maintenance of body weight in patients with CHF.

For the economic evaluation we will measure resource utilization and calculate costs which are to be related to health outcomes measured in quality adjusted life years (QALYs). There are no adverse effects known to be caused by this kind of intervention. Nevertheless, all severe events (unplanned hospital admission, visit at GP, nursing home placement, out-of-hospital death) will be monitored regularly.

3. STUDY DESIGN

The study is designed as a single-centre randomized two-armed parallel group trial. All patients will receive a baseline assessment after giving their informed consent. Those who are subsequently randomized to the intervention group receive the intervention (see figure 1) starting with the initial session shortly before discharge. All patients will be contacted by phone after 3, 6, and 9 months in order to document a core set of relevant outcome measures. After one year a final investigation will take place in the hospital or at home.

Figure 1: Study design

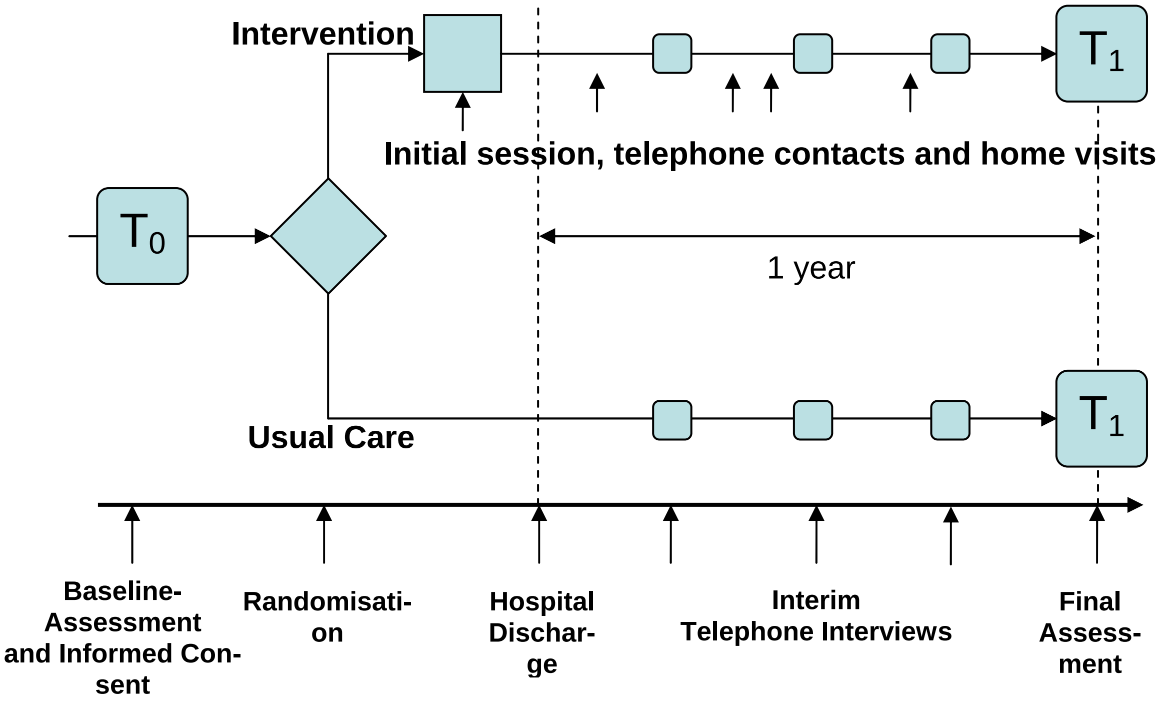

3.1 STUDY OBJECTIVES

3.1.1 Primary objective

The primary objective is to assess whether a case management intervention by trained nurses can reduce readmission or out of hospital death in aged patients with myocardial infarction.

3.1.2 Secondary objectives

Secondary objectives are the estimation of the cost-utility ratio of this case management intervention and to compare case management vs. routine care with respect to secondary outcomes such as compliance with medication.

For patients with specific comorbidity, additional outcome criteria will be compared between the two groups, e.g. adjustment of blood glucose values and hemoglobin-A_{1c} in patients with type 2 diabetes, and maintenance of body weight in patients with CHF.

3.2 INTERVENTION AND CONTROL GROUP

3.2.1 Discharge

The study intervention will consist of an initial information session which will take place one or two days before hospital discharge. In this session the patient will be provided with infor-

mation material about the disease (and comorbidities), about medication and with behavioural recommendations (nutrition, physical activity, smoking etc.).

Furthermore, a first home visit will be arranged (within one to two weeks after discharge), if accepted by the patient, otherwise an appointment for a telephone call will be made. If possible, close relatives of the patient will also be informed in detail and may participate in the discharge session.

The GP of the patient will be informed about the study participation at discharge by telephone through the study physician.

If a patient is going to receive a cardiac rehabilitation after discharge, the first home visit or first telephone call will be arranged within one to two weeks after completion of the rehabilitation.

The medication at discharge will not be influenced by the intervention. In case of problems with medication, the study nurse will contact the GP of the patient after the first contact with the patient.

3.2.2 Telephone contacts

Telephone calls (at least every 3 months) and home visits (1 to 4) will be carried out according to patient need. The patients will primarily be responsible themselves to carry out the planned activities, and the study nurse will only support the patients. If the patient needs further assistance or advices, the study nurses will be achievable for the patient in the daytime by telephone on weekdays.

3.2.3 Home visits

The first home visit will be scheduled to take place 7 to 14 days after discharge. If the patient has a stay in a rehabilitation hospital immediately after discharge from the Augsburg hospital, the first home visit will be postponed accordingly. The most important elements of the home visits will be: to detect problems or risks, to give advice and to refer to the general practitioner, if necessary. Risk assessment will be done according to four prespecified risk categories. The higher the group level the more contacts (telephone and home visits) will be arranged by the study nurse. The risk level will be determined by the compliance, the social network, and the comorbidity. The duration of the visit should be between 60 and 90 minutes. At the first home visit patients will be instructed how the prescribed drugs have to be taken and what would happen in the case of non-compliance with medication. Furthermore, to patients with diabetes advice will be given regarding nutrition and physical activity, and patients with CHF information will be encouraged to regular weight control. During the visits, measurements of blood glucose, blood pressure, and weight will be performed.

3.2.4 Training and supervision of study nurses

In August 2008, the training of the three study nurses and the study physician took place over several days.
The following topics and diseases were intensively discussed with the study nurses:

- Coronary heart disease/myocardial infarction/angina pectoris
- Heart failure
- Treatment of myocardial infarction
- Risk factors of cardiovascular diseases
- Action of particular drugs
- Diabetes mellitus
- Psychological factors and myocardial infarction

Furthermore, the structure and the contents of the “Heart book” handed out to the patients in the intervention group at discharge were discussed.
The general course of the study and the practical implementation of the study modules were trained.

Both study doctor and study nurses received a training of patient recruitment (identification of eligible patients, inclusion and exclusion of patients, communication between study nurses and study doctor).

The study doctor received a training on patient information and obtaining informed consent. Furthermore, the procedure of the clinical examination and personal interview was trained as well as the contact with office-based doctors.

Moreover, study nurses were trained on the following issues:

- Personal interviews with the patients in the clinic
- Assessment of patients’ records
- Home visits (making appointments, interview, and examination)
- Telephone interviews and supervision of patients in the intervention group
- Training on the intervention modules (medication compliance, social support, change of life style, blood pressure control, blood sugar, body weight, involvement of the general practitioner)
- Training on data entry and documentation as well as handling of the database
- Training on the module “Health Care” including the assessment of the EQ-5D
- Training on entry of medication with IDOM and the data collection with CATI

3.2.5 Cooperation with GPs

Before the study begins all GPs and cardiologists in the study region will be informed by mail about the study. The president of the local association of physicians supports the study. Additionally a press release about the study will be made.

At discharge, the study nurse or the study physician will contact the patient’s GP if there is need for action, providing the patient has given consent and willingness.

3.2.6 Treatment in control group

The control group will receive usual care. Patients can use or apply for all available services in the area. In both groups, patients may be subscribed to a disease management program (DMP) if their general practitioner participates in a DMP of their statutory health insurance company. In addition, they may use out-patient nursing services during the follow-up period.

However, patients who already receive out-patient nursing services at time of recruitment will be excluded from the study.

3.3 PATIENT SELECTION

3.3.1 Inclusion criteria

In the planned study, all patients of the age group 70 years and older with a first or recurrent myocardial infarction during the recruitment period (September 2008 to August 2009) who are treated in the Augsburg Hospital should be included.

The Augsburg Hospital is the largest hospital in the region of Augsburg offering a coronary care unit as well as coronary angiography and angioplasty facilities 24 hours a day. All myocardial infarctions according to ESC and ACC criteria will be included [25].

3.3.2 Exclusion criteria

Patients who already live in institutionalized care or who are already receiving regular support by ambulatory care services will be excluded. In addition, patients who already plan to move into institutional care or outside the study region within the next months will be excluded as well.

Patients with severe comorbidity (e.g. terminal cancer) which makes rehospitalization within the next months necessary or is associated with a life expectancy of less than one year will be excluded.

Patients who are not able to communicate in German language will be excluded.

Patients who are unable or unwilling to give written informed consent (e.g. patients with dementia) cannot be included in the study.

All consecutive patients screened for eligibility will be documented in a patient log with anonymous data on age, gender, comorbidity, and reasons for ineligibility.

3.4 OUTCOME MEASURES

3.4.1 Primary endpoint

The main endpoint of readmission will be measured as time between initial hospital discharge to first unplanned readmission to hospital (at least for 24 hours). Out of hospital death from any cause will also be counted as an event.

3.4.2 Secondary endpoints

Baseline data on all recruited patients (intervention and control group) will be collected at the index hospitalization, usually one or two days before the patient will be discharged. Data on sociodemographic and health status characteristics, functional status, and psychosocial status are collected by a specially trained study nurse. After discharge, standardized telephone interviews with the patients, and, if reasonable, with the patient's general practitioner, are planned at 3, 6, and 9 months after index hospital discharge identifying patient's readmissions, acute care visits to physicians, clinics, and ambulatory departments. Data on functional status and quality of life will be also collected during these interviews. In the interven-

tion group additional telephone calls as well as home visits will take place as part of the intervention. Additional information will be documented on these opportunities in order to monitor process quality.

Twelve months after the index hospitalization all patients in both groups who are still alive will be examined. For this purpose, the patients are visited at home or are examined in hospital. This follow-up examination includes among other things a personal interview, the assessment of functional status (in cooperation with SP3), the collection of data on mental status and quality of life (in cooperation with SP4), adherence to medication, health care utilization, the measurement of blood pressure and body weight as well as the collection of a blood sample to determine parameters such as blood glucose, HbA1c, blood count, and lipid parameters.

Cost data as well as quality of life data will be collected at baseline, at interim telephone contacts, and during the final assessment.

3.5 METHODS AGAINST BIAS

3.5.1 Randomization

All patients meeting the inclusion criteria and giving informed consent will be randomly assigned to the intervention or control group. The allocation ratio will be 1:1. To ensure the concealment of the allocation, randomisation will be provided per telephone call to the biostatistical center at the Helmholtz Zentrum München, German Research Center for Environmental Health, where a randomization list is kept. A minimization procedure will be used which tries to achieve balanced treatment groups with respect to gender, age (70-79 vs. 80+), and comorbidities (diabetes and CHF).

3.5.2 Blinding

Blinding of participants will not be possible, because home visits will only be offered in the intervention group. However, observer blindness will be maintained in the final assessment after 12 months.

3.6 PROPOSED SAMPLE SIZE / POWER CALCULATIONS

3.6.1 Primary statistical hypothesis

To make the sample size calculation more transparent, it is based on a chi-squared test comparing the probabilities to be readmitted within one year after discharge. The final statistical analysis will use the Cox model (which is equivalent to the log rank test) for the comparison of readmission times, which has a slightly higher power than the chi-squared test.

3.6.2 Relevant difference and error probabilities

Based on a similar study by Young and colleagues [26] with patients 70 years of age and our own experience from the pilot study, we expect an event rate (readmission or out-of-hospital death) of 40% in the control group. If the study is designed to have 80% power or more to detect an improved rate of 25% in the intervention group (i.e. Δ=0.15) at a two-sided type I error level of 5%, at least 152 patients per group will be needed (27, p. 10ff.).

3.6.3 Rate of loss to follow up

We expect that the drop-out rate does not exceed 10% during the 1-year follow-up period. Because the included persons will be at advanced age, often living alone and without intensive social contact, it can be assumed that the planned regular home visits by study nurses as well as telephone contacts will be well accepted by the participants. In order to allow for loss to follow-up (patient withdrawing consent or moving away from study region), we plan to recruit a total of 338 patients for the trial.

3.6.4 Required sample size

If the study is designed to have 80% power or more to detect an improved rate of 25% in the intervention group (i.e. Δ=0.15) at a two-sided type I error level of 5%, at least 152 patients per group will be needed (27, p. 10ff.).

3.7 FEASIBILITY OF RECRUITMENT

3.7.1 Previous experiences

An observational follow-up study (AMI Elderly) in the same age cohort of patients with acute MI has been performed in the same institution. Between March 2005 and March 2006 all patients with an acute myocardial infarction (aged 75 to 84 years) treated in the Augsburg Hospital were recruited (N=260). From all acute MI patients surviving at least 24 hours detailed information from each patient was abstracted from the charts through specially trained nurses. After a follow-up period of about 12 months all of the patients who were still alive were examined. For this purpose, the patients were visited at home. Follow-up data were gathered by a personal interview and a physical examination. This follow-up examination includes among other things the assessment of functional status, the collection of data on depression, adherence to medication, health care utilization, the measurement of blood pressure, and collection of a blood sample. For all deceased patients death certificates were obtained from the local health departments. The procedures in this study are identical in many aspects to the control arm of the planned randomized study.

3.7.2 Achievability of recruitment rate

From a previous study in the same hospital (see pilot study detailed above) with similar inclusion criteria but without randomization we know that about 250 to 300 patients in the age range 75 to 84 years will be available per year. Thus, the estimated number of patients aged 70+ surely will be achievable.

We expect that only a small proportion of patients will refuse randomization. Patients will be informed that both groups will have telephone contact over one year but differ with respect to intensity of contact. The final assessment after 12 months will be presented as an extra incentive for all patients.

4. STATISTICAL ANALYSES

4.1 ANALYSIS OF PRIMARY ENDPOINT

The statistical analysis for the primary endpoint will focus on the null hypothesis that there is no difference between intervention and control with respect to the distribution of time to unplanned readmission (including out-of-hospital death). This hypothesis will be statistically tested using a Cox proportional hazards model. We will include the three covariates which are included in the balanced randomization algorithm (age, gender, and comorbidity) in the Cox model and test the intervention parameter with a two-sided significance level of α=0.05.

4.2 INTERIM ANALYSIS

We will not perform a pre-planned interim analysis because recruitment will be finished until enough data have been collected. However, events in both groups will be monitored regularly and in case of striking differences the Data Monitoring and Safety Committee (DMSC) will be informed. Subgroup analyses will be performed, but only in an exploratory context.

4.3 ANALYSIS OF SECONDARY ENDPOINTS

Statistical analyses for secondary outcomes will be based on (generalized) linear models depending on the type and distribution of the outcome variable. Since these will be multiple tests for which control of the overall error level cannot be achieved, these will be regarded as exploratory analyses.

4.4 MISSING DATA/ANALYSIS SETS

The primary population for statistical analysis under the intention-to-treat (ITT) approach comprises of all randomized patients (ITT analysis set).

For the time-to-event endpoint, those patients who refuse any further participation in the trial or who are lost-to-follow up will be censored at the time of last contact (unless they have already been rehospitalized before that time).

Losses to follow-up will be treated differently in the analysis according to whether they are unwilling or unable to show up for the final examination or whether they withdraw their participation and request the deletion of their personal data. In the first case, we will be able to assess readmission from physician or hospital records and thus include the patient in the primary analysis. In the second case, the patient will enter the primary analysis as a censored observation. For secondary analyses requiring follow-up data at the final one year assessment patients lost to follow-up will not be available. Sensitivity analyses based on imputation techniques will be used to judge whether study results could be influenced by loss to follow-up.

4.5 ECONOMIC ANALYSIS

Data for the economic analysis will come from different sources. In this group of aged patients, cost diaries may not be feasible. Therefore cost data will be collected via patients. Table 1 summarizes the data collection for the economic evaluation.

Table 1: Overview on the cost categories, the measurement of the resource use and its valuation

Cost category	Resource use	Valuation
Outpatient care	Number of consultations	€ per consultation
Prescribed drugs	Central pharmaceutical number, ATC code, dose rate	Pharmacy retail price
Inpatient care	Length of stay and number of days on the intensive care unit, if possible hospital data	Daily hospital rate, if applicable DRG
Rehabilitation	Length of stay	Daily rehabilitation rate
Remedies (physiotherapy, massage)	Number of visits	Average rates
Ambulatory care	Days per week and hours per day	Average rates per hours
Home help	Hours per week	Average labor costs for a home help
Informal care	Care level declared by the long term care insurance	Max amount paid by the long term care insurance if the patient does not make use of formal care

All cost data will be retrospectively assessed by the patient. As valid information on the resource use can only be retrospectively gathered for a short previous time period the assessment will be conducted quarterly. Due to the wide range and prices of remedies and aids available we only ask for the sum of treatments in case of remedies and do not assess aids. Within the last years, standardized instruments measuring the resource use were developed such as the RAI (Resident Assessment Instrument [27] or the RUD (Resource utilization of dementia) [28]. They all assess the relevant cost categories retrospectively. On the basis of these questionnaires as well as of our experiences gathered within several KORA studies we developed a questionnaire for the patients that can be used in personal and telephone interviews.
The valuation of the resource use will be based on the valuation rates calculated by Krauth et al. (2005) [29]. As the valuation rates are based on data for 2000 the calculations will be updated.
Especially in the economic evaluation of interventions with the elderly informal care plays an important role. However, as the assessment is very time consuming and the interview time should be minimized we refrained from assessing informal care time. To estimate the cost of informal care we use the information of the existence of a care level declared by the long term care insurance. If the patient receives allowance for nursing care and does not receive formal care, it is assumed that the patient has an informal caregiver. In that case, the maximum amount for care at home paid by the long term care insurance will be used as cost for informal care.

Cost for the intervention excluding the costs caused by the study will be considered. Cost components will include labor costs, travel expenses, telephone costs etc.

In order to calculate QALYs, the EQ-5D questionnaire (five items) will be applied at baseline, at interim telephone contacts, and during the final assessment. Additionally, the VAS will be applied at baseline and final assessment. It is planned to assess the EQ-5D also in case of readmission to the Augsburg hospital. However, as it is not possible that the study team will be informed about readmissions the EQ-5D will only be assessed if the study team detects the readmission. Therefore, the assessment of the questionnaire is only for exploratory analyses.

It is planned to analyse the study in form of a cost-utility-analysis from the societal perspective. This means that incremental costs of the intervention (vs. control) are set in relation to the number of quality-adjusted life years (QALYs) gained. The resulting incremental cost-effectiveness ratio (ICER) is an international standard for reporting results of economical evaluation studies. In addition, we will calculate additional costs per life year saved and per year without event.

For the estimated ICER value a 95% confidence interval will be calculated based on bootstrap methods.

4.6 ANALYSIS PLAN

A detailed statistical analysis plan will be provided before the start of the analysis.

5. ETHICAL CONSIDERATIONS

5.1 RANDOMIZATION

The randomized study is regarded as ethically feasible, since the control group receives usual care whereas the intervention group will receive an additional nurse-based case management support which may be beneficial, but still needs to be evaluated. Specific risks of case management interventions have not been reported in the literature.

5.2 INFORMED CONSENT

Prior to the inclusion into the study, each patient will be informed by the treating physician about the aim and the design of the study, the expected benefits and possible risks that may occur. The patient information form is written and printed in a way suitable for elderly patients (see Appendix). Patients will be required to give their written consent prior to inclusion into the study (Consent Form see Appendix). Patients can withdraw from the study at any time without any disadvantage.

5.3 DATA CONFIDENTIALITY

Data confidentiality will be guaranteed and the data management concept will be reviewed and agreed by the responsible data protection commissioners of the Augsburg Hospital and the Helmholtz Zentrum München.

5.4 ETHICAL APPROVAL

The study will be conducted in accordance to the Declaration of Helsinki and to the Guidelines for Good Clinical Practices (GCP). The study protocol, patient information, and the Informed Consent Form have been approved by the Ethics Committee of the Bayerische Landesärztekammer (BLÄK).

5.5 PROTOCOL AMENDMENTS

Changes in the study protocol may be advised by the Steering Committee and have to be added to the study protocol in form of written amendments. If an amendment may affect patient safety, the relevant ethics committee will be consulted.

6. TRIAL MANAGEMENT

6.1 STUDY PROCEDURES

6.1.1 Recruitment

All patients in the Augsburg Central Hospital will be screened at least every other day for newly admitted patients with myocardial infarction. All suspected cases above 70 years of age are documented in a log file. If a patient was admitted to another ward than cardiology the patient will be transferred to the cardiology on the same or the next day.

All eligible patients will be informed about the study and asked to give written consent to participate in the study by a study physician. In order to check eligibility and ability to give informed consent, a dementia screening test will be performed in addition to clinical judgment. This information will take place usually 3 to 5 days before planned discharge.

6.1.2 Randomization

If the patient agrees to participate, the study physician will complete the baseline documentation form and register the patient at the randomization office via phone call (Monday to Friday between 9 a.m. and 5 p.m.) or email or fax. The following information will be required for registration:

- Name of the physician or study nurse
- Initials of the patient
- Gender
- Birth date
- History of diabetes
- History of CHF

The randomization office will inform the study physician about the randomly selected treatment either directly within the telephone call or within one day in case of registration by email or fax.

6.1.3 Baseline Assessment

Baseline data on all recruited patients (intervention and control group) will be collected at the index hospitalization, usually one or two days before the patient will be discharged. Data on sociodemographic and health status characteristics, functional status, and psychosocial status are collected by a specially trained study nurse. Clinical interview and physical examinations will be performed by a study physician. Documentation and assessments include:

- Activities of daily living (ADL): Barthel Index and HAQ-DI
- Instrumental activities of daily living (IADL): IADL scale developed by Lawton/Brody
- Timed up and go (TUG)
- Hand grip strength measurement
- Mini Mental State Test (MMSE)
- SCREEN II, Version II (Nutrition)
- EQ-5D
- WHO-Five Well-being Index
- F-SozU
- Geriatric depression Scale (GDS)
- Items on vision, hearing, chewing and swallowing from Geriatric Assessment (Lachs)
- Baseline questionnaire including items on multimorbidity and physical examination
- Health care utilization

The Barthel Index uses ten variables describing activities of daily living (ADL) and mobility. A higher number is associated with a greater likelihood of being able to live at home [30].

The HAQ-DI assesses ADL and instrumental ADL as an indicator for chronic diseases and its long-time impact [33].

The Instrumental Activities of Daily Living Scale (IADL) is an appropriate instrument to assess independent living skills. This instrument is intended to be used among older adults, and can be used in community or hospital settings [31].

The timed get up and go test is a measurement of mobility. It includes a number of tasks such as standing from a seating position, walking, turning, stopping, and sitting down which are all important tasks needed for a person to be independently mobile. The instrument is widely employed in the examination of elders [32].

Hand grip strength is a common clinically used strength capacity measure. It has been shown in epidemiological studies to be related to leg extension strength and to be associated to recurrent falling in daily life [35].

The MMSE is a brief screening test (range from 0 to 30) that assesses several cognitive functions and is one of the most widely used screening instruments for cognitive impairment [36].

The SCREEN II, Version II, is a multidimensional method of nutritional evaluation that allows the diagnosis of malnutrition and the change in personal diet [37].

The EQ-5D is a generic instrument in which respondents are asked to rate their current health state. This instrument allows calculating quality-adjusted life years [38].

The WHO-Five Well-being Index covers positive mood (good spirits, relaxation), vitality (being active and waking up fresh and rested), and general interests (being interested in things) [39].

The GDS measures depression and has been tested and used extensively with the older population. The GDS may be used with healthy, medically ill and mild to moderately cognitively impaired older adults. It has been extensively used in community, acute and long-term care settings [40].

The F-SozU indicates the possibility to receive social support as perceived by the patient in case of need [41].

At baseline physical examinations will be performed including height, weight, blood pressure measurement, auscultation of lung and heart. Furthermore, multimorbidity will be assessed.

Health care utilization is also described in point 4.5.

6.1.4 Interim Assessment

After discharge, standardized telephone interviews with the patients, and, if reasonable, with the patient's general practitioner, are planned at 3, 6, and 9 months after index hospital discharge identifying patient's readmissions, unscheduled acute care visits to clinics and emergency departments. Data on functional status, quality of life, and health care utilization will be also collected during these interviews. In the intervention group additional telephone calls as well as home visits will take place as part of the intervention. Additional information will be documented on these opportunities in order to monitor process quality. Regular quarterly telephone interviews will be preceded by a letter stating the date and time of the call and including a short questionnaire.

6.1.5 Final assessment

Twelve months after the index hospitalization all patients in both groups who are still alive will be examined. For this purpose, the patients are visited at home or are examined in hospital. This follow-up examination includes among other things a personal interview, the assessment of functional status, the collection of data on mental status and quality of life, adherence to medication, health care utilization, the measurement of blood pressure and body weight as well as the collection of a blood sample to determine parameters such as blood glucose, HbA1c, blood count, and lipid parameters.

Table 2: Measuring intervals

Category	Instrument	Assessment by	T_0	T_3	T_6	T_9	T_{12}
Anamnesis	Baseline questionnaire	Physician	●				
Anamnesis	Geriatric Assessment	Physician	●				
Quality of life	EQ-5D	Patient	●	●	●	●	●
Quality of life	WHO Well Being Test	Patient	●				●
Cognition	MMSE	Physician	●				●
ADL	Barthel Index	Physician	●				●
IADL	Lawton/Brody	Physician	●				●
Functioning	HAQ-DI	Patient	●				●
Functioning	Grip strength measure-ment	Physician	●				●
Mobility	Time up and go	Physician	●				●
Social Sup-port	F-sozU	Patient	●				●
Nutrition	Screen II, Version II	Patient	●				●
Depression	GDS	Patient	●				●
Resource use	Questionnaire	Patient	●	●	●	●	●

6.2 QUALITY ASSURANCE/MONITORING

Quality standards of the KORA Myocardial Infarction Registry and of central KORA data and study management in Neuherberg will be applied to this trial. An external quality assurance board will be convened which monitors the quality of all projects within the network throughout the field phase by site visits and regular quality assurance reports. Quality of the standard operating procedures (SOP) for the field study will be prepared prior to the study's participant recruitment. These SOPs will be reviewed and approved by the external quality assurance board. The accumulated study data will be regularly reviewed and evaluated for study conduct, progress, and efficacy by an internal quality assurance staff. This staff will regularly provide reports about data quality, completeness, and recruitment efforts. Based on the reports of the internal quality assurance staff, the external quality assurance board will make recommendations concerning the improvement of data quality and will also regularly perform quality assurance reports.

6.3 DATA MANAGEMENT

Data management in the KORINNA study has a local component in the Augsburg Myocardial Infarction Registry as part of the Augsburg Hospital and a central component in the Helmholtz Zentrum München located in Neuherberg.

6.3.1 Data management in Augsburg

The local data management is focussed on organisation of patient recruitment and follow-up and on data entry. It consists of the following four data bases:

6.3.1.1 Organisation-DB

This is an Adabas/Natural application where patient contact data (name, address) are stored together with the patient ID. It is used to administrate appointment dates for patient examinations, home visits, telephone calls etc. Only here patient identifying data are stored, whereas in all other applications the patient ID is used as pseudonym.

6.3.1.2 ACCESS data base

Most data are documented on case report forms during personal interview. These data will be stored in a separate data base after double data entry.

Furthermore, relevant data referring to dropout will be assessed.

6.3.1.3 CATI-data base

This is an ACCESS application for computer assisted telephone interviews programmed with the DAIMON software, a development by the Helmholtz Zentrum München. The following data are entered with this instrument:

- Module “Health Care” as part of the regular telephone interviews in the intervention and control group
- Additional telephone interviews in the intervention group
- Module “Health Care” as part of the baseline interview (double entry from paper forms)
- Home visit documentation (double entry from paper forms)

6.3.1.4 KORA Myocardial Registry data base

The participants of the KORINNA study receive a copy of the interview and of the documents stored in the KORA Myocardial Registry data base. Since 2009, all myocardial patients aged 85 or younger will be assessed in the registry. Only those myocardial patients study older than 85 are usually not included in the registry. However, those patients of the KORINNA they will be interviewed and their data will be assessed in the registry database.

6.3.2 Data transfer

Personalized data of the organisation DB are only accessible in the study center. However, aggregated data can be provided to the KORINNA team of the HMGU for statistical analyses (e. g. number of home visits and telephone calls etc.).

The data will be sent monthly to the IGM at the HMGU.

Data will be sent within the data transfer of the KORA Myocardial Registry to the Institute of Epidemiology at the HMGU which takes place in sporadic intervals.

For quality assurance the data will be sent to the IGM at the HMGU in monthly intervals.

All data will be assigned to the patient ID and will be sent via Email.

6.3.3 Data security

Data backup in the Helmholtz Zentrum München is performed daily by automatical routines.

6.3.4 Archiving

The principal investigator and the data management center will retain all relevant study documents, including the patient consent forms, the ethics committee approval, for a minimum of 10 years beyond the end of the study.

The pseudomized patient data will be stored in the study data base for at least 10 years after the end of the study.

6.5 TRIAL TIMELINE FLOW

Milestones	Year 1 (2008)												Year 2 (2009)												Year 3 (2010)												Year 4 (2011)			
Study Preparation						x	x	x																																
Recruitment									x	x	x	x	x	x	x	x	x	x	x	x	x	x	x																	
Treatment									x	x	x	x	x	x	x	x	x	x	x	x	x	x	x	x	x	x	x	x	x	x	x	x	x	x	x					
1-Year-Assessment																					x	x	x	x	x	x	x	x	x	x	x	x	x	x	x					
Data entry and Quality control									x	x	x	x	x	x	x	x	x	x	x	x	x	x	x	x	x	x	x	x	x	x	x	x	x	x	x	x				
Analysis & Reporting																																		x	x	x	x	x	x	

6.6 TRIAL TERMINATION

The trial shall be terminated prematurely if study monitoring shows that recruitment falls clearly short of the calculated sample size. A decision for early termination will only be taken after careful review of the situation by the Data Safety and Monitoring Board.

7. RESPONSIBILITIES AND TRIAL SUPPORT

7.1 TRIAL-SUPPORTING FACILITIES

The trial will have local support from the KORA Augsburg Myocardial Infarction Registry which is located at the Augsburg Hospital. Based on standardized procedures the register team routinely identifies all hospitalized patients in the age range 25 to 74 years with clinically suspected myocardial infarctions since 1984. In the framework of the planned intervention study the register staff will also identify the persons with myocardial infarction age 75+. The standardized proceeding and the long standing experience guarantees that all patients with acute myocardial infarctions will be captured. Furthermore, the KORA Myocardial Infarction Registry will make workrooms, telephone sets, and PC's available to the co-workers of the intervention study. In addition, the car of the register staff will be available for the planned

home visits. Furthermore, the equipment for the examination after 12 months will be provided by the Augsburg Hospital and the KORA Study Center.

The Institutes of Epidemiology (EPI) and of Health Economics and Health Care Management (IGM) at the Helmholtz Zentrum München, German Research Center for Environmental Health provide a common infrastructure for study coordination, data management and quality control of all KORA studies.

7.2 STEERING COMMITTEE

The steering committee of the KORINNA study will comprise of the principal investigators and Prof. von Scheidt, Prof. Wichmann and Prof. Leidl.

7.3 DATA SAFETY AND MONITORING BOARD

An independent Data Safety and Monitoring Board (DSMB) has been nominated to review the data in case that the continuous study monitoring shows any unexpected events. Its members are Prof. Wolfgang Koenig (University of Ulm Medical Center), Prof. Hans-Helmut König (University of Leipzig), and Prof. Hans J. Trampisch (University of Bochum).

This DMSB will approve the study protocol and SOP, with regard to subject safety, recruitment, randomisation, intervention, data management, quality control, and analysis. It should identify the relevant parameters and the format of the information to be regularly reported. The DMSB will review the data related to efficacy, recruitment, randomization, compliance, retention, protocol adherence, SOPs, form completion, intervention effects, inclusion criteria, and subject safety and should make a recommendation for or against the trial's continuation.

7.4 DISSEMINATION AND PUBLICATION OF RESULTS

The publication of the study results in international as well as national peer reviewed journals is self-evident. If a case management program in aged patients after an MI would be cost-saving, such a programme should be implemented into the local, regional, and national structures of the German health system. The finding of the planned intervention study should be used to guide clinicians, administrators and policymakers in the provision of high-quality care to older patients with CHD and comorbidities.

In Germany, there already exist out-patients nursing-services, which visit patients based on their needs of care. However, such a care is limited to those persons classified as “in need of care” after examination through a co-worker of the nursing care insurance. Thus, at the moment out-patients nursing services are not straightened to case management. However, it might be possible to use these services as case managers for multimorbid patients too, to avoid readmissions, physical disability, and admission to a nursing home.

8. REFERENCES

[1] Zeymer U, Gitt A, Winkler R, Zahn R, Junger C, Schiele R, Gottwik M, Senges J. [Mortality of patients who are older than 75 years after ST elevation myocardial infarction in clinical practice]. Dtsch Med Wochenschr 2005; 130:633-6.
[2] Botkin NF, Spencer FA, Goldberg RJ, Lessard D, Yarzebski J, Gore JM. Changing trends in the long-term prognosis of patients with acute myocardial infarction: a population-based perspective. Am Heart J 2006; 151:199-205.
[3] Bush DE, Ziegelstein RC, Tayback M, Richter D, Stevens S, Zahalsky H, Fauerbach JA. Even minimal symptoms of depression increase mortality risk after acute myocardial infarction. Am J Cardiol 2001; 88:337-41.
[4] Kupari M, Lindroos M, Iivanainen AM, Heikkila J, Tilvis R. Congestive heart failure in old age: prevalence, mechanisms and 4-year prognosis in the Helsinki Ageing Study. J Intern Med 1997; 241:387-94.
[5] Rask-Madsen C, Jensen G, Kober L, Melchior T, Torp-Pedersen C, Hildebrand P. Age-related mortality, clinical heart failure, and ventricular fibrillation in 4259 Danish patients after acute myocardial infarction. Eur Heart J 1997; 18:1426-31.
[6] Bell DS. Diabetic cardiomyopathy. Diabetes Care 2003; 26:2949-51.
[7] Rodriguez-Artalejo F, Guallar-Castillon P, Banegas Banegas JR, del Rey Calero J. Trends in hospitalization and mortality for heart failure in Spain, 1980-1993. Eur Heart J 1997; 18:1771-9.
[8] Haldemann G, Croft J, Giles W, Rashidee A. Hospitalization of patients with heart failure: National Hospital Discharge Survey, 1985-1995. Am Heart J 1999; 137:352-60.
[9] Gooding J, Jette AM. Hospital readmissions among the elderly. J Am Geriatr Soc 1985; 33:595-601.
[10] Gijsen R, Hoeymans N, Schellevis FG, Ruwaard D, Satariano WA, van den Bos GA. Causes and consequences of comorbidity: a review. J Clin Epidemiol 2001; 54:661-74.
[11] Ashton CM, Kuykendall DH, Johnson ML, Wray NP, Wu L. The association between the quality of inpatient care and early readmission. Ann Intern Med 1995; 122:415-21.
[12] Petrie MC, Berry C, Stewart S, McMurray JJ. Failing ageing hearts. Eur Heart J 2001; 22:1978-90.
[13] Clark AM, Hartling L, Vandermeer B, McAlister FA. Meta-analysis: secondary prevention programs for patients with coronary artery disease. Ann Intern Med 2005; 143:659-72.
[14] Lim WK, Lambert SF, Gray LC. Effectiveness of case management and post-acute services in older people after hospital discharge. Med J Aust 2003; 178:262-6.
[15] Preen DB, Bailey BE, Wright A, Kendall P, Phillips M, Hung J, Hendriks R, Mather A, Williams E. Effects of a multidisciplinary, post-discharge continuance of care intervention on quality of life, discharge satisfaction, and hospital length of stay: a randomized controlled trial. Int J Qual Health Care 2005; 17:43-51.
[16] McAlister FA, Lawson FM, Teo KK, Armstrong PW. Randomised trials of secondary prevention programmes in coronary heart disease: systematic review. BMJ 2001; 323:957-62.
[17] Naylor MD, Brooten D, Campbell R, Jacobsen BS, Mezey MD, Pauly MV, Schwartz JS. Comprehensive discharge planning and home follow-up of hospitalized elders: a randomized clinical trial. JAMA 1999; 281:613-20.
[18] Ihl R, Weyerer S. Forschungsaktivitäten zur Hochaltrigkeit unter besonderer Berücksichtigung der Demenz. In: Familie Bf, editor. Vierter Bericht zur Lage der älteren Generation. Berlin, 2002:60-9.
[19] Lee PY, Alexander KP, Hammill BG, Pasquali SK, Peterson ED. Representation of elderly persons and women in published randomized trials of acute coronary syndromes. JAMA 2001; 286:708-13.
[20] Pfisterer M. Long-term outcome in elderly patients with chronic angina managed invasively versus by optimized medical therapy: four-year follow-up of the randomized Trial of Invasive versus Medical therapy in Elderly patients (TIME). Circulation 2004; 110:1213-8.

[21] Weir RA, McMurray JJ. Epidemiology of heart failure and left ventricular dysfunction after acute myocardial infarction. Curr Heart Fail Rep 2006; 3:175-80.
[22] Balzi D, Barchielli A, Buiatti E, Franceschini C, Mangani I, Del Bianco L, Monami M, Valente S, Gensini GF, Marchionni N. Age and comorbidity in acute myocardial infarction: a report from the AMI-Florence Italian registry. Am J Geriatr Cardiol 2006; 15:35-41.
[23] Simpson E, Pilote L. Quality of life after acute myocardial infarction: a comparison of diabetic versus non-diabetic acute myocardial infarction patients in Quebec acute care hospitals. Health Qual Life Outcomes 2005; 3:80.
[24] Stewart S, Marley JE, Horowitz JD. Effects of a multidisciplinary, home-based intervention on unplanned readmissions and survival among patients with chronic congestive heart failure: a randomised controlled study. Lancet 1999; 354:1077-83.
[25] Lowel H, Meisinger C, Heier M, Hormann A. The population-based acute myocardial infarction (AMI) registry of the MONICA/KORA study region of Augsburg. Gesundheitswesen 2005; 67 Suppl 1:S31-7.
[26] Young W, Rewa G, Goodman SG, Jaglal SB, Cash L, Lefkowitz C, Coyte PC. Evaluation of a community-based inner-city disease management program for postmyocardial infarction patients: a randomized controlled trial. CMAJ 2003; 169:905-10.
[27] Ljunggren G, Morris J. The Resident Assessment Instrument (RAI) and resource use in dementia care. In: Wimo A JB, Karlsson G, Winblad B, editor. Health Economics of Dementia. Chichester: John Wiley & Sons, 1998.
[28] Wimo A, Wetterholm AL, Mastey V, Winblad B. Evaluation of the Healthcare Resource and Caregiver Time in Anti-Dementia Drug Trials - A Quantitative Battery. In: Wimo A, Jönsson B, Karlsson G, Winblad B, editors. Health Economics of Dementia. Chichester: John Wiley & Sons, 1998:465-99.
[29] Krauth C, Hessel F, Hansmeier T, Wasem J, Seitz R, Schweikert B. [Empirical standard costs for health economic evaluation in Germany -- a proposal by the working group methods in health economic evaluation]. Gesundheitswesen 2005; 67:736-46.
[30] Mahoney FI, Barthel DW. Functional Evaluation: The Barthel Index. Md State Med J 1965; 14:61-5.
[31] Lawton MP, Brody EM. Assessment of older people: self-maintaining and instrumental activities of daily living. Gerontologist 1969; 9:179-86.
[32] Podsiadlo D, Richardson S. The timed "Up & Go": a test of basic functional mobility for frail elderly persons. J Am Geriatr Soc 1991; 39:142-8.
[33] Bruhlmann P, Stucki G, Michel BA. Evaluation of a German version of the physical dimensions of the Health Assessment Questionnaire in patients with rheumatoid arthritis. J Rheumatol 1994, 21:1245-9.
[34] Enright PL, McBurnie MA, Bittner V, Tracy RP, McNamara R, Arnold A, Newman AB. The 6-min walk test: a quick measure of functional status in elderly adults. Chest 2003; 123:387-98.
[35] Stel VS, Smit JH, Pluijm SM, Lips P. Balance and mobility performance as treatable risk factors for recurrent falling in older persons. J Clin Epidemiol 2003; 56:659-68.
[36] Folstein MF, Folstein SE, McHugh PR. "Mini-Mental State": a practical method for grading the cognitive state of patients for the clinician. Journal of Psychiatric Research 1975; 12:189-98.
[37] Keller HH, Goy R, Kane SL. Validity and reliability of SCREEN II. Seniors in the community: risk evaluation for eating and nutrition, Version II. Eur J Clin Nutr. 2005 Oct., 59:1149-57.
38] The EuroQol Group. EuroQol--a new facility for the measurement of health-related quality of life. The EuroQol Group. Health Policy 1990; 16:199-208.
[39] Bonsignore M, Barkow K, Jessen F, Heun R. Validity of the five-item WHO Well-Being Index (WHO-5) in an elderly population. Eur Arch Psychiatry Clin Neurosci 2001; 251 Suppl 2:II27-31.
[40] Yesavage JA, Brink TL, Rose TL, Lum O, Huang V, Adey M, Leirer VO. Development and validation of a geriatric depression screening scale: a preliminary report. J Psychiatr Res 1982-83; 17:37-49.
[41] Fydrich T, Sommer G, Tydecks S, Brähler E. Fragebogen zur sozialen Unterstützung (F-SozU): Normierung der Kurzform (K-14). Z Med Psychol 2009, 18:43-48.
